# Microneedle-based drug and vaccine delivery via nanoporous microneedle arrays

**DOI:** 10.1007/s13346-015-0238-y

**Published:** 2015-06-05

**Authors:** Koen van der Maaden, Regina Luttge, Pieter Jan Vos, Joke Bouwstra, Gideon Kersten, Ivo Ploemen

**Affiliations:** MyLife Technologies, Enschede, The Netherlands; Leiden Academic Centre for Drug Research, division of Drug Delivery Technology, Leiden University, Leiden, The Netherlands; Institute for Translational Vaccinology (Intravacc), Bilthoven, The Netherlands

**Keywords:** (Trans)dermal drug delivery, Microneedles, Porous microneedles

## Abstract

In the literature, several types of microneedles have been extensively described. However, porous microneedle arrays only received minimal attention. Hence, only little is known about drug delivery via these microneedles. However, porous microneedle arrays may have potential for future microneedle-based drug and vaccine delivery and could be a valuable addition to the other microneedle-based drug delivery approaches. To gain more insight into porous microneedle technologies, the scientific and patent literature is reviewed, and we focus on the possibilities and constraints of porous microneedle technologies for dermal drug delivery. Furthermore, we show preliminary data with commercially available porous microneedles and describe future directions in this field of research.

## Introduction

The skin is an attractive organ for drug and vaccine delivery because it is easily accessible and has a large surface area that is available for drug administration, and dermal drug delivery is potentially pain-free [1–4]. Furthermore, drug delivery via the skin circumvents the first-pass effect of the liver, swallowing problems, inconvenient injections, and drug absorption/stability problems in the gastrointestinal tract [1, 5–7]. Besides, the skin contains a large number of antigen-presenting cells (i.e., Langerhans cells and dermal dendritic cells) and is therefore a suitable site for vaccination [8, 9]. However, for transdermal delivery, only about 20 active drug molecules are on the market, all of them being low-molecular-weight drugs, illustrating that it is difficult to overcome the skin barrier [10]. This barrier, i.e., the stratum corneum, complicates foreign compounds, including drugs, from entering the body. To overcome the stratum corneum barrier, several drug delivery systems have been developed. One of the most promising delivery systems are microneedles, which are needle-like structures with a length of less than 1 mm that are used to deliver drugs into the skin in a minimally-invasive and potentially pain free manner [1, 4, 8].

**Fig. 1:**
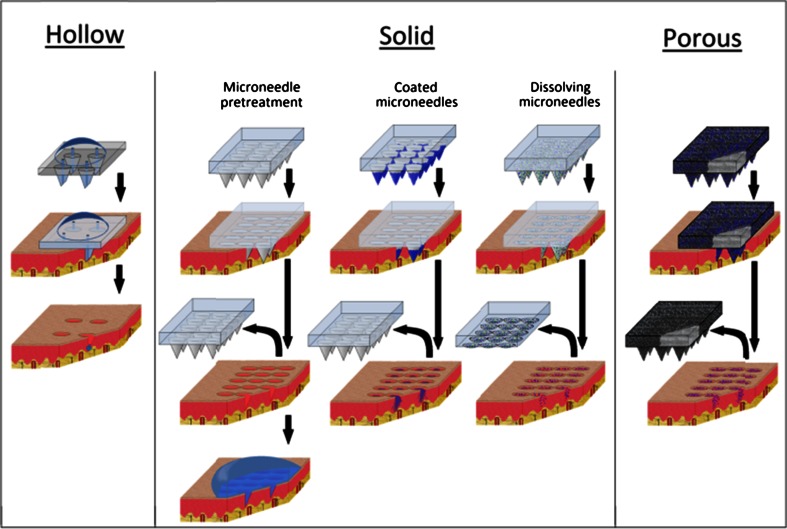
Microneedle-based drug delivery via hollow, solid, and porous microneedles. Image adapted from [1]

In the literature, two types of microneedles (hollow and solid) have been extensively described for drug delivery via four delivery approaches (Fig. 1), as reviewed elsewhere [11–13] and briefly described below. Drug delivery via hollow microneedles (a miniaturized form of hypodermic needles) is achieved by pressing a liquid drug formulation through the bore of the microneedle into the skin [14–22]. Drug delivery via solid microneedles is achieved by three technologies. For drug delivery via microneedle pretreatment, microneedles are first pierced into the skin and subsequently, a patch with a drug formulation is applied onto the site of microneedle application, leading to diffusion of the drug into the skin [23–34]. For drug delivery via coated microneedles, the microneedle surface is first coated with a drug. Upon piercing of the skin, the drug coating is hydrated and detaches from the microneedle surface, resulting in delivery of the drug into the skin [35–48]. For drug delivery via dissolving microneedles, a drug is entrapped in microneedles made of a soluble material. Upon piercing, the microneedles dissolve through hydration, leading to the delivery of the drug into the skin [49–59]. Each of these types of delivery approaches has its particular merits and disadvantages with respect to drug delivery applications [1]. Another type of microneedles for dermal drug delivery only received minimal attention in the microneedle field: porous microneedles. Hence, only little is known about drug delivery via these microneedles. This review focuses on the possibilities and constraints of porous microneedle technologies for dermal drug delivery, shows preliminary data with commercially available porous microneedles, and describes future directions in this field of research.

## Drug delivery via porous microneedle technologies

Dermal drug delivery via porous microneedles differs in several aspects from the delivery approaches via solid microneedles, although there are also similarities. Porous microneedle arrays are envisaged as a single-unit-drug delivery system, whereby the whole microneedle array (i.e., the microneedles and potentially the backplate) contains either a liquid or a dry drug formulation.

- In case of a liquid formulation: a drug formulation will be first loaded into the pores of a microneedle array. Subsequently, the microneedles are pierced into the skin, and the drug diffuses from the microneedle matrix into the skin. As the microneedles are depleted of the drug, the drug diffuses from the drug reservoir (microneedle backplate) via the microneedles into the skin. Hence, drug delivery via porous microneedles has elements of microneedle pretreatment (i.e., drug delivery is a diffusion-based process), with the major technical difference that porous microneedles remain inside the skin during drug delivery.
- In case of a dry formulation: a drug formulation will be loaded into the pores of the microneedles and dried, e.g., by heat, vacuum, or freeze drying. Upon piercing into the skin, the drug formulation must be hydrated with interstitial fluid (ISF). Hydration can occur via capillary forces of the pores. Consequently, the dry formulation dissolves and the drug diffuses from the microneedle pores into the skin, as described above.

Several patents describe porous microneedles that may potentially be used for drug delivery and/or sampling of biological fluids [60–68]. However, so far, only a few research papers have been published on porous microneedles [69–74]. Among them, one publication reports on the delivery of a model drug (antibody) into the skin via porous microneedles [73], and another publication reports on the dermal delivery of a red hydrophobic dye [74]. Furthermore, porous microneedles have, to our knowledge, not yet been (successfully) applied for dermal immunization, apart from the very rudimentary results on ovalbumin-antibody responses in the serum of mice in a small scale animal experiment [75].

In brief, several platform technologies for porous microneedles exist, utilizing porous silicon [62, 64–66, 68], colloidal silica [67], polymers [64], and ceramics (e.g., calcium phosphate [63], gypsum, brushite [74, 76], and alumina [60, 72]), as described in detail below. These microneedle arrays are either completely [60, 62, 64, 66, 72] (i.e., the microneedles including the backplate) or partially (i.e., only the microneedle tip) made of a porous material [65, 69]. Alternatively, solid microneedle arrays may be coated with a porous material [61, 63, 66].

### Porous polymeric microneedles

Porous microneedles made of poly lactic acid (PLA), a biodegradable polymer, may have several advantages. Microneedles made of dissolving/biodegradable materials have the advantage that broken microneedles left inside the skin will eventually disappear. Thus far, porous PLA microneedles have been made with a porosity of 75 %. However, these microneedles lacked strength and were consequently unable to penetrate skin [70]. Therefore, this microneedle technology requires improvements, e.g., by decreasing the porosity to increase the microneedle strength, to become fit for application in dermal drug delivery in its present form.

### Porous silicon microneedles

Porous silicon is generally made by electrochemical etching of plain silicon. Porous silicon is a non-toxic and biocompatible material that has been produced with a large variety of pore sizes (1 nm to 10 μm) [77–79] and is, depending on the porosity and pore size, biodegradable into silicic acid [78–80]. Whereas high porosity silicon (> 70 % porosity) is completely biodegradable [78] (e.g., complete dissolution of a 68–70 % porous silicon (40–60 nm pores) implant occurs within 8 weeks in vivo [79]), low porosity silicon and macroporous silicon (> 50 nm pores) are quite bioinert materials similar to normal silicon [78]. Silicon is already a brittle material and making it porous its strength is reduced (e.g., inducing 40 % porosity into silicon leads to a tenfold decreased mechanical strength [81]). Hence, porous silicon microneedles may easily break and stay inside the skin upon piercing. Therefore, porous silicon microneedles should preferably be biodegradable (thus have a porosity of >70 % and a pore size of <50 nm). Another disadvantage of porous silicon is that it cannot be stored under ambient conditions because of the limited stability. Degradation (oxidation) naturally occurs over time and accelerates with increasing moist content, at increasing pH values, and elevated temperature (thermal oxidation) [78–80]. Porous microneedles have been produced from several types of silicon [35, 82–84]. However, the pore morphology (i.e., pore size, orientation, and shape) upon electrochemical etching is dependent on the type of silicon [77–79]. Therefore, the introduction of pores into silicon microneedles will not be a general strategy for producing porous microneedles. Besides, introducing pores lead to a decreased sharpness in silicon microneedles [69], which could result in a decreased penetration ability of these microneedles. Finally, the direct manufacture of microneedles in silicon is relatively expensive in terms of the starting material as well as the involved MEMS processing steps compared to microneedle production technologies based on microreplication.

In conclusion, porous silicon microneedles may be applicable for drug delivery if produced as high porosity, small pore-sized silicon. Therefore, this strategy might be limited to dermal delivery of low-molecular-weight drugs and small therapeutic peptides.

### Microneedles coated with a porous ceramic material

Microneedles with a solid core and a porous shell have been made by electrochemical coating of solid metal microneedles (thus having a solid core and porous shell) with calcium phosphate, a bioceramic [71]. This has the advantage over completely porous microneedles that the microneedles (the core) retain their strength and thereby allow insertion of the microneedles into the skin [1]. Another advantage of this specific approach is the large pore size (0.2–0.5 μm), which enables most protein-based and subunit vaccines to be loaded into the pores of the microneedles. However, the porous calcium phosphate coating onto microneedles lacks strength, i.e., when these microneedles are inserted into the skin in the dry state (i.e., no liquid is loaded in the pores), the calcium phosphate coating breaks off into or onto the skin [71]. This is especially undesirable in the skin because calcium phosphate is a second generation bioceramic (i.e., it is a bioactive material) that promotes cell adhesion [85, 86] and could thereby lead to skin irritation. Although porous calcium phosphate is a material that is biodegradable via osteoclastic activity (resorption) and dissolution [85–87], it takes a long time before the material is degraded, e.g., the weight loss of porous calcium in a physiological buffer was about 6 % after 90 days [88].

### Dissolving porous ceramic microneedles

Recently, dissolving porous ceramic microneedles have been made from self-setting ceramics (gypsum (CaS) and brushite (CaP)) by microreplication techniques. The advantage of this system is that these self-setting dissolving ceramic microneedles have an increased mechanical strength as compared to polymeric or sugar-based dissolving microneedles. Besides, the pore size and thereby the drug release profile can be tweaked. These microneedles were used for the delivery of a red hydrophobic dye in porcine skin by applying dye-coated self-setting ceramic microneedles onto the skin. Besides, zolpidem tartrate (307 Da) loaded self-setting ceramic microneedles released up to 70 % of their content after 48 h in a cellulose matrix in vitro. However, the drug loading circumstances for these microneedles are unfavorable for proteins. Drug loading is either performed during the molding process, requiring 0.5 M citric acid (brushite) or involves an exothermic reaction (gypsum), or by adding the drug dissolved in ethanol post hoc [74, 76]. Therefore, the applicability of these microneedles is likely limited to the delivery of low-molecular-weight drugs.

### Porous alumina microneedles

Alumina (Al_2_O_3_) is a first generation bioceramic (i.e., this material has a good chemical stability and is almost bioinert) [85, 89–91]. Alumina ceramics have good mechanical strength as compared to monocrystalline silicon (fracture toughness of 3.75–4.85 MPa·m^1/2^ [92] and 0.83·–0.94 MPa·m^1/2^ [93], respectively). Furthermore, alumina has been used for implants (e.g., dental implants, bone implants) [89–91, 94]. Porous alumina structures can be made with pores varying from 10 nm up to hundreds of micrometers [89–91]. As the microneedle sharpness is an important factor for skin penetration [1] (e.g., microneedles with a tip diameter of <50 nm may be reproducibly inserted into skin upon manual application, whereas microneedles with a tip diameter of 1–5 μm may require an impact-insertion applicator, to ensure sufficient skin penetration, depending on the microneedle length and density), the alumina particles that are used for producing porous ceramic microneedles should be sufficiently small to fully fill the desired mold structure and large enough to produce the target pore size. Microneedles with a very small pore size will limit the applicability of the porous alumina microneedle technology for the delivery of biomacromolecular drugs such as biologicals and vaccines. Recently, microneedles with an average pore size of 80 nm have been made from alumina nanoparticles with an average particle diameter of 300 nm [72, 73]. This resulting pore size enables in theory the loading of small molecules, (therapeutic) peptides, proteins, drugs (e.g., antibodies, cytokines), and (subunit) vaccines, while the microneedle still retains a sufficient tip sharpness. These microneedles were successfully used to deliver an antibody into ex vivo human skin [73], which holds promise for their use as a macromolecular drug delivery system.

Since the reported data on porous microneedles in dermal drug delivery are limited to a few studies, we performed in this work in vitro studies with ceramic (alumina) nanoporous microneedle arrays (npMNA). These preliminary studies as described in Characterization of ceramic porous microneedle arrays in vitro provide guidance for future investigations into the applicability of porous ceramic microneedles in dermal drug delivery.

## Characterization of ceramic porous microneedle arrays in vitro

### Materials and methods

#### Materials

Fluorescein, trypan blue, and fluorescently labeled nanoparticles of 30 nm (carboxylate-modified, fluorescent yellow-green, λ_ex_ 470 nm/λ_em_ 505 nm), 50 nm (amine-modified, fluorescent blue, λ_ex_ 360 nm/λ_em_ 420 nm), and 100 nm (sulfate-modified, fluorescent orange, λ_ex_ 520 nm/λ_em_ 540 nm) were purchased from Sigma Aldrich. PBS (pH 7.2) was obtained from Invitrogen, and AKP30 alumina particles were obtained from Sumitomo Chemical.

#### Pore volume determination

To determine the pore volume, 24 npMNAs (prepared as previously described [72]) were first loaded with fluorescein by totally immersing (dipping) them in a 0.1-mg/mL fluorescein in PBS (pH 7.2) solution. Subsequently, surface adsorbed liquid was removed by using pressurized nitrogen, and the arrays were subsequently incubated in 10 mL release buffer (PBS) on a shaking device at 500 rpm. After 1 h, a time point ensuring 100 % release of the fluorescein from the npMNAs, the concentration fluorescein in the release buffer was determined, from which the pore volume was calculated. The concentration fluorescein was determined by using a calibration curve (4–250 ng/mL) and measuring the fluorescence on a Synergy™ Mx (Bio-Tek) microplate reader in a black 96-well plate with an excitation wavelength of 494 nm and an emission wavelength of 521 nm.

#### Loading and release of a small compound

To determine the release of a low-molecular-weight model drug from npMNAs, three arrays (with a weight of ±80 mg per array) were loaded with 500 ng fluorescein by applying a drop of 5 μL 0.1 mg/mL fluorescein in PBS (pH 7.2) solution onto the microneedles, which was absorbed within a few seconds. Next, the arrays were incubated in 10 mL PBS on a shaking device at 500 rpm, and the released fluorescein was determined at several time points (1–30 min) as described above.

#### Loading and release of nanoparticles

To investigate the size limit of molecules that can be loaded into the npMNAs, the loading and release performance of fluorescently labeled nanoparticles with a size of 30, 50, and 100 nm was investigated. First, the pore volume (V_pore_) of each of the used npMNAs was determined as described above. Subsequently, nanoparticles were loaded into the npMNAs by incubating them in a vial on a shaking device at 500 rpm in a loading volume (V_loading_) of 100 μL, containing 10 mg/mL (1 % solids) nanoparticles in PBS. As a control a vial with 100 μL of a-10 mg/mL nanoparticle dispersion without a npMNA was used. After 15 min, the concentration of nanoparticles in the vials with (C_MN_) and without (C_contr._) a npMNA was determined by using a calibration curve and measuring the specific fluorescence of the fluorescently labeled nanoparticles. Subsequently, the amount of loaded nanoparticles and the loading efficiency (LE, the fraction of the pore volume that is available for nanoparticle entrapment) were calculated according to Eqs. 1 and 2, respectively.

1 $$ \mathrm{Loaded}\ \mathrm{N}\mathrm{P}\mathrm{s} = \left({\mathrm{V}}_{\mathrm{loading}} \times {\mathrm{C}}_{\mathrm{contr}.}\right) - \left(\left[{\mathrm{V}}_{\mathrm{loading}} - {\mathrm{V}}_{\mathrm{pore}}\right] \times {\mathrm{C}}_{\mathrm{MN}}\right) $$

2 $$ \mathrm{L}\mathrm{E} = \left(\mathrm{Loaded}\ \mathrm{N}\mathrm{P}\mathrm{s}\right)/\ \left({\mathrm{V}}_{\mathrm{pore}} \times {\mathrm{C}}_{\mathrm{contr}.}\right) \times 100\% $$

Next, the extent of liquid was removed from the microneedle arrays by using pressurized nitrogen, and released amount of nanoparticles was determined after the arrays were incubated in 10 mL release buffer (PBS) for 2 h on a shaking device at 500 rpm. The release efficiency was defined as the percentage released nanoparticles of the loaded nanoparticles.

#### Microneedles and skin penetration

**Fig. 2:**
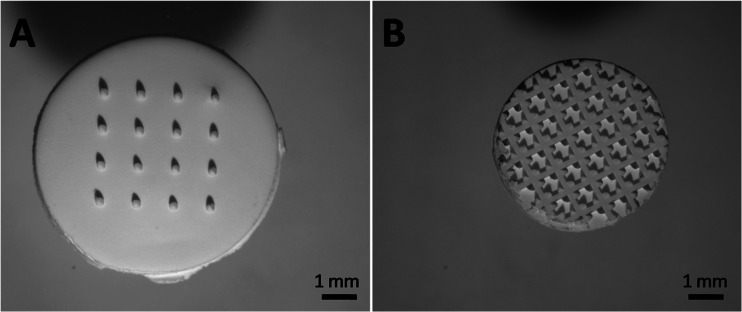
Nanoporous ceramic microneedle arrays (MyLife Technologies) containing 16 microneedles with a length of 370 μm (**a**) and 126 microneedles with a length of 200 μm (**b**)

The penetration ability of npMNAs in ex vivo human skin was investigated by using empty npMNAs with two different geometries, as supplied by MyLife Technologies, containing either 16 microneedles with a length of 370 μm (Fig. 2a) or 126 microneedles with a length of 200 μm per array (Fig. 2b). The zeta potential of the microneedle material (alumina) was measured in 0.1 × PBS (pH 7.2) on a Zetasizer Nano (Malvern Instruments). Ex vivo abdomen human skin was obtained from local hospitals within 24 h after cosmetic surgery and was dermatomed to a thickness of 600 μm. Subsequently, the microneedles were placed onto the skin, and the skin was pierced by applying an impact-insertion applicator onto the microneedles with a velocity of 3 m/s [95]. This procedure was repeated five times with both microneedle arrays. Next, a drop of 70 μL Trypan blue was applied for 1 h, after which the skin was washed once with 70 % ethanol and twice with PBS. Finally, the stratum corneum was removed by tape-stripping (Scotch tape) as previously described [34].

### Results and discussion

#### Pore volume

**Fig. 3:**
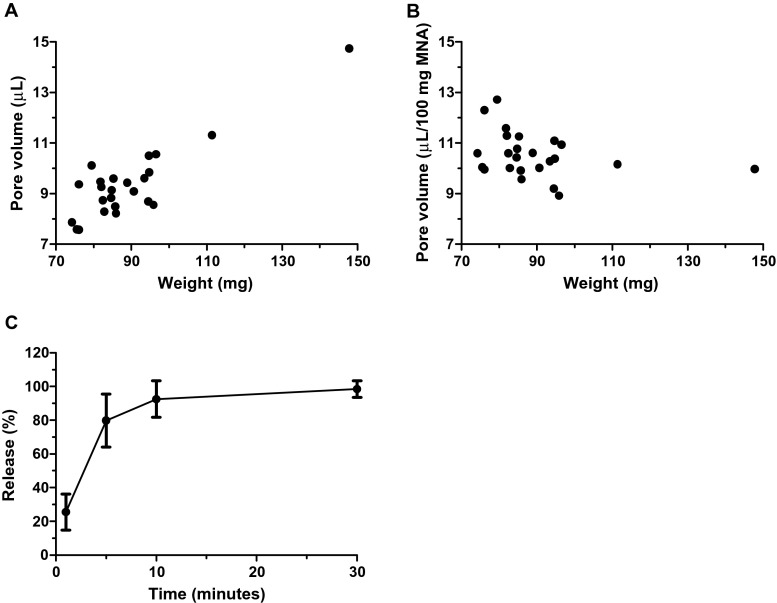
Pore volume as a function of weight of 24 individual microneedle arrays (**a**) and the normalized pore volume (**b**). Release of fluorescein (mean ± SD, *n* = 3) from 500 ng fluorescein loaded nanoporous microneedle arrays in PBS with a weight of ±80 mg/array (**c**)

The pore volume is an important factor for the amount of drugs that can be loaded into porous microneedle arrays. Therefore, the pore volume of npMNAs with varying weights was determined, as shown in Fig. 3a. Besides, Fig. 3b shows that npMNAs have a reproducible pore volume (10.52 ± 0.88 μL/100 mg microneedle array, mean ± SD, *n* = 24). This implies that these microneedle arrays, having an average density of 3.84 g/cm^3^, have a porosity of 40.41 ± 3.40 % (mean ± SD, *n* = 24), which is comparable to reported values [73].

#### Release of a small molecule

npMNAs (±80 mg/array) loaded with 5 μL of a 0.1-mg/mL fluorescein solution efficiently released their contents within 30 min upon incubation in 10 mL PBS (Fig. 3c), which represents a fast release for low-molecular-weight drugs in vitro. However, in case of a dermal application, the surface of the microneedles that is inside the skin determines how fast small molecules diffuse from the microneedle arrays into the skin, because molecules can only diffuse from the microneedle surface that is pierced into the skin. There are several product-related factors that determine the rate of drug delivery, e.g., the solubility and concentration of the drug molecule, the thickness of the backplate, and the properties of the microneedle array itself (e.g., length, sharpness, porosity, strength, surface area, density [34, 35, 70]). However, the rate of drug delivery is also dependent on variables more difficult to control: the quality of the penetration (i.e., the depth of microneedle insertion and the penetration efficiency), the manner of microneedle application (e.g., applied force, time of application, usage of an applicator [34, 96]), and on the type of skin (e.g., elasticity and subcutaneous fat/dermis/stratum corneum thickness that varies by age, gender, race, illness, genetic factors, body weight, etc. [97–100]). To gain more insight into the usability of the npMNAs for drug release into the skin, diffusion studies should be performed.

#### Loading and release of nanoparticles

**Fig. 4:**
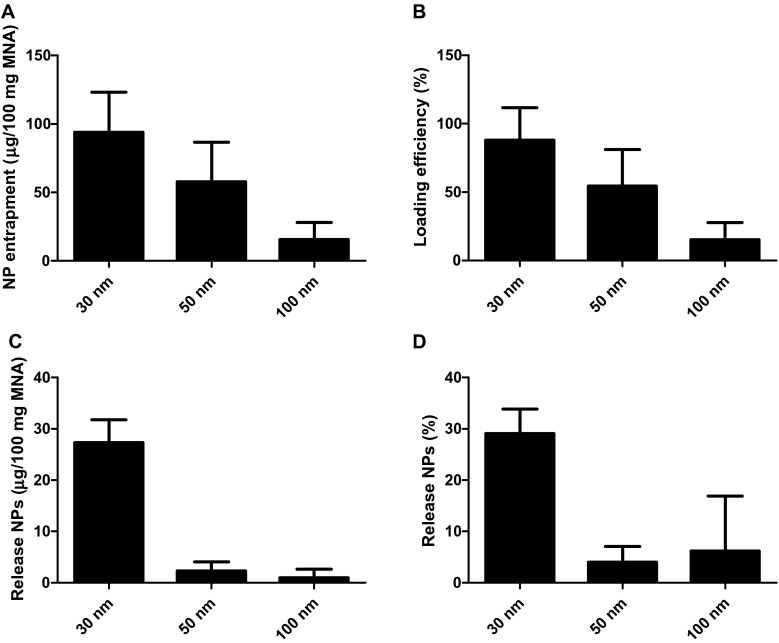
Loading and release of fluorescently labeled nanoparticles (NPs) into nanoporous ceramic microneedle arrays (npMNAs). The amount of loaded nanoparticles into npMNAs (**a**), and the loading efficiency (**b**). The amount of released nanoparticles after 2 h in PBS at room temperature from nanoparticle-loaded npMNAs (**c**), and the released nanoparticles expressed in percentage (**d**). Results are represented as mean ± SD, *n* = 3

In order to have a size range in which proteins and vaccines can be loaded into npMNAs, fluorescently labeled nanoparticles with different sizes were used. As represented in Fig. 4a, this led to a loading of 94, 58, and 16 μg for 30, 50, and 100 nm nanoparticles, respectively, showing that the total amount of nanoparticles that was loaded into the npMNAs (±80 mg/array) was dependent on the nanoparticle size. As shown in Fig. 4b, 30 nm nanoparticles (88 %) and 50 nm nanoparticles (54 %) were efficiently loaded into the npMNAs. Besides, even nanoparticles of 100 nm were loaded into the npMNAs albeit with a substantially lower loading efficiency of 15 %. Next, the amount of released nanoparticles from nanoparticle-loaded npMNAs was determined, showing that 27 (29 %), 2 (4 %), and 1 μg (6 %) of the 30, 50, and 100 nm nanoparticles were released, respectively (Fig. 4c, d). However, the release of the 50 nm nanoparticles was relatively low, which could be due to adsorption of the nanoparticles to the microneedle inner surface, which is likely a result of the nanoparticle charge, i.e., the 30 nm and 100 nm are negatively charged, and the 50 nm nanoparticles are positively charged, whereas alumina is negatively charged (zeta potential of −18 mV at pH 7.2).

In conclusion, these data show that particles with a size up to 100 nm can be loaded into the npMNAs, although not very efficiently for the 100 nm nanoparticles. Both the loading and the release efficiency are dependent on the particle size and likely on the surface charges. Nevertheless, the experiments with these model compounds indicate that a large variety of drugs, including proteins and vaccines can be potentially loaded and released from the npMNAs. To gain more insight into the usability of the npMNAs for drug delivery, loading of biomacromolecules and vaccines with different sizes, geometries, and charges should be investigated.

#### Skin penetration

**Fig. 5:**
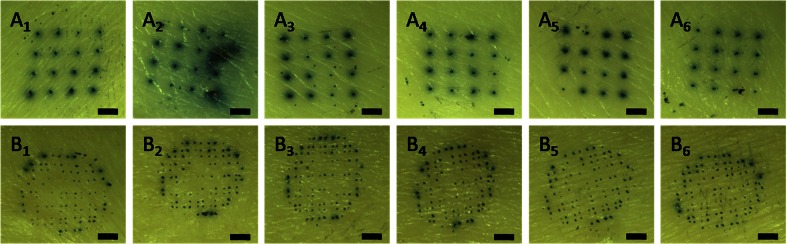
Six subsequent penetrations of ex vivo human skin by a ceramic nanoporous microneedle array (1–6 respectively), that contained 16 microneedles with a length of 370 μm (**a**) or 126 microneedles with a length of 200 μm (**b**). The *size bar* represents 1 mm

The penetration of skin is essential to enable dermal drug delivery through the use of (porous) microneedles. The efficiency and reproducibility of microneedle insertion affects the rate of drug delivery, e.g., more and deeper penetrations will lead to a faster diffusion/delivery of the drug. Therefore, the penetration ability of npMNAs was investigated (Fig. 5). As shown in Fig. 5a, it was observed that empty npMNAs, containing 16 microneedles with a length of 370 μm, which were applied six times, were efficiently (100 %) and reproducibly inserted into ex vivo human skin. Besides, no visible breakage of the microneedles was observed, as also observed by all microneedles penetrating the skin after the sixth application. The npMNAs containing 126 microneedles with a length of 200 μm also efficiently and reproducibly (84.5 ± 3.6 %; mean ± SD, *n* = 6) penetrated ex vivo human skin (Fig. 5b). Also for these npMNAs, no visible breakage of the microneedles was observed after six applications. From the first to the sixth application, 108, 103, 101, 114, 105, 108 spots (reflecting penetrated microneedles) were counted, respectively, showing that these npMNAs can efficiently and reproducibly penetrate human skin.

## Future directions and perspectives

In this study, ceramic npMNAs have shown to be efficiently and reproducibly loaded with small molecules as well as nanoparticles. Moreover, results show the release of a small molecule and nanoparticles up to 100 nm in vitro. Furthermore, we have shown that npMNAs are able to efficiently and reproducibly pierce ex vivo human skin. Besides, we did not observe breakage of the microneedles. Although alumina is a biocompatible and bioinert material, it is not biodegradable. Therefore, the next step is to investigate how skin reacts when ceramic npMNAs are applied in vivo. It is also envisaged to implement ceramic npMNAs with an application device that facilitates efficient and reproducible microneedle insertion. Currently, various therapeutic model compounds are tested in combination with the ceramic npMNAs using a human skin explant model. Furthermore, several antigens with different sizes (e.g., tetanus, diphtheria, hepatitis B, polio, and influenza) are currently under investigation for their loading ability into the ceramic npMNAs and the subsequent release in vitro and into ex vivo human skin. Future studies shall elucidate more details of the pharmacodynamics as well as the pharmacokinetic response upon utilizing porous microneedles for drug and vaccine delivery. In conclusion, npMNAs may have potential for future microneedle-based drug and vaccine delivery and could be a valuable addition to the other microneedle-based drug delivery approaches.

## References

[1] van der Maaden K, Jiskoot W, Bouwstra J (2012). Microneedle technologies for (trans)dermal drug and vaccine delivery. J Control Release.

[2] Haq MI, Smith E, John DN, Kalavala M, Edwards C, Anstey A (2009). Clinical administration of microneedles: skin puncture, pain and sensation. Biomed Microdevices.

[3] Kaushik S, Hord AH, Denson DD, McAllister DV, Smitra S, Allen MG (2001). Lack of pain associated with microfabricated microneedles. Anesth Analg.

[4] Kim Y-C, Park J-H, Prausnitz MR (2012). Microneedles for drug and vaccine delivery. Adv Drug Deliv Rev.

[5] Ball AM, Smith KM (2008). Optimizing transdermal drug therapy. Am J Health Syst Pharm.

[6] Prausnitz MR, Gill HS, Park J-H. Microneedles for drug delivery, In: Modified release drug delivery. 2008, pp. 295-309.

[7] Tanner T, Marks R (2008). Delivering drugs by the transdermal route: review and comment. Skin Res Technol.

[8] Bal SM, Ding Z, van Riet E, Jiskoot W, Bouwstra J (2010). Advances in transcutaneous vaccine delivery: do all ways lead to Rome?. J Control Release.

[9] Nicolas J-F, Guy B (2008). Intradermal, epidermal and transcutaneous vaccination: from immunology to clinical practice. Expert Rev Vaccines.

[10] Subedi RK, Oh SY, Chun M-K, Choi H-K (2010). Recent advances in transdermal drug delivery. Arch Pharm Res.

[11] Quinn HL, Kearney M-C, Courtenay AJ, McCrudden MT, Donnelly RF (2014). The role of microneedles for drug and vaccine delivery. Expert Opin Drug Deliv.

[12] Cheung K, Das DB. Microneedles for drug delivery: trends and progress, Drug Deliv. DOI: 10.3109/10717544.2014.986309 (2014) 1-17. 10.3109/10717544.2014.986309 25533874

[13] Indermun S, Luttge R, Choonara YE, Kumar P, Toit LC, Modi G (2014). Current advances in the fabrication of microneedles for transdermal delivery. J Control Release.

[14] Bodhale DW, Nisar A, Afzulpurkar N (2010). Structural and microfluidic analysis of hollow side-open polymeric microneedles for transdermal drug delivery applications. Microfluid Nanofluid.

[15] Davis SP, Martanto W, Allen MG, Prausnitz MR (2005). Hollow metal microneedles for insulin delivery to diabetic rats. IEEE Trans Biomed Eng.

[16] Gardeniers HJGE, Luttge R, Berenschot EJW, Boer MJ, Yeshurun SY, Hefetz M (2003). Silicon micromachined hollow microneedles for transdermal liquid transport. J Microelectromech Syst.

[17] Gupta J, Felner EI, Prausnitz MR (2009). Minimally invasive insulin delivery in subjects with type 1 diabetes using hollow microneedles. Diabetes Technol Ther.

[18] Martanto W, Moore JS, Kashlan O, Kamath R, Wang PM, O'Neal JM (2006). Microinfusion using hollow microneedles. Pharm Res.

[19] McAllister DV, Wang PM, Davis SP, Park J-H, Canatella PJ, Allen MG (2003). Microfabricated needles for transdermal delivery of macromolecules and nanoparticles: fabrication methods and transport studies. Proc Natl Acad Sci.

[20] Mukerjee EV, Collins SD, Isseroff RR, Smith RL (2004). Microneedle array for transdermal biological fluid extraction and in situ analysis. Sensors Actuators A Phys.

[21] van der Maaden K, Trietsch B, Kraan H, Varypataki EM, Romeijn S, Zwier R (2014). Novel hollow microneedle technology for depth controlled microinjection-mediated dermal vaccination: a study with polio vaccine in rats. Pharm Res.

[22] Wang PM, Cornwell M, Hill J, Prausnitz MR (2006). Precise microinjection into skin using hollow microneedles. J Investig Dermatol.

[23] Bal SM, Caussin J, Pavel S, Bouwstra JA (2008). In vivo assessment of safety of microneedle arrays in human skin. J Control Release.

[24] Bal SM, Kruithof AC, Zwier R, Dietz E, Bouwstra JA, Lademann J (2010). Influence of microneedle shape on the transport of a fluorescent dye into human skin in vivo. J Control Release.

[25] Bal SM, Slütter B, Jiskoot W, Bouwstra JA (2011). Small is beautiful: N-trimethyl chitosan-ovalbumin conjugates for microneedle-based transcutaneous immunisation. Vaccine.

[26] Bal SM, Slütter B, Riet E, Kruithof AC, Ding Z, Kersten GFA (2010). Efficient induction of immune responses through intradermal vaccination with N-trimethyl chitosan containing antigen formulations. J Control Release.

[27] Chabri F, Bouris K, Jones T, Barrow D, Hann A, Allender C (2004). Microfabricated silicon microneedles for nonviral cutaneous gene delivery. Br J Dermatol.

[28] Henry S, McAllister DV, Allen MG, Prausnitz MR (1998). Microfabricated microneedles: a novel approach to transdermal drug delivery. J Pharm Sci.

[29] Kalluri H, Kolli CS, Banga AK (2011). Characterization of microchannels created by metal microneedles: formation and closure. AAPS J.

[30] Li X, Zhao R, Qin Z, Zhang J, Zhai S, Qiu Y (2010). Microneedle pretreatment improves efficacy of cutaneous topical anesthesia. Am J Emerg Med.

[31] Slütter B, Bal SM, Zhi D, Jiskoot W, Bouwstra JA (2011). Adjuvant effect of cationic liposomes and CpG depends on administration route. J Control Release.

[32] Verbaan FJ, Bal SM, van den Berg DJ, Groenink WHH, Verpoorten H, Lüttge R (2007). Assembled microneedle arrays enhance the transport of compounds varying over a large range of molecular weight across human dermatomed skin. J Control Release.

[33] Banks SL, Paudel KS, Brogden NK, Loftin CD, Stinchcomb AL (2011). Diclofenac enables prolonged delivery of naltrexone through microneedle-treated skin. Pharm Res.

[34] van der Maaden K, Sekerdag E, Jiskoot W, Bouwstra J (2014). Impact-insertion applicator improves reliability of skin penetration by solid microneedle arrays. AAPS J.

[35] van der Maaden K, Yu H, Sliedregt K, Zwier R, Leboux R, Oguri M (2013). Nanolayered chemical modification of silicon surfaces with ionizable surface groups for pH-triggered protein adsorption and release: application to microneedles. J Mater Chem B.

[36] van der Maaden K, Varypataki EM, Romeijn S, Ossendorp F, Jiskoot W, Bouwstra J (2014). Ovalbumin-coated pH-sensitive microneedle arrays effectively induce ovalbumin-specific antibody and T-cell responses in mice. Eur J Pharm Biopharm.

[37] Gill HS, Prausnitz MR (2007). Coated microneedles for transdermal delivery. J Control Release.

[38] Gill HS, Soderholm J, Prausnitz MR, Salberg M (2010). Cutaneous vaccination using microneedles coated with hepatitis C DNA vaccine. Gene Ther.

[39] Kim Y-C, Quan F-S, Compans RW, Kang S-M, Prausnitz MR (2010). Formulation of microneedles coated with influenza virus-like particle vaccine. AAPS PharmSciTech.

[40] Matriano JA, Cormier M, Johnson J, Young WA, Buttery M, Nyam K (2002). Macroflux microprojection array patch technology: a new and efficient approach for intracutaneous immunization. Pharm Res.

[41] Pearton M, Kang S-M, Song J-M, Kim Y-C, Quan F-S, Anstey A (2010). Influenza virus-like particles coated onto microneedles can elicit stimulatory effects on Langerhans cells in human skin. Vaccine.

[42] Widera G, Johnson J, Kim L, Libiran L, Nyam K, Daddona PE (2006). Effect of delivery parameters on immunization to ovalbumin following intracutaneous administration by a coated microneedle array patch system. Vaccine.

[43] Zhang Y, Brown K, Siebenaler K, Determan A, Dohmeier D, Hansen K (2011). Development of lidocaine-coated microneedle product for rapid, safe, and prolonged local analgesic action. Pharm Res.

[44] Zhu Q, Zarnitsyn VG, Ye L, Wen Z, Gao Y, Pan L (2009). Immunization by vaccine-coated microneedle arrays protects against lethal influenza virus challenge. Proc Natl Acad Sci.

[45] Chen X, Prow TW, Crichton ML, Jenkins DWK, Roberts MS, Frazer IH (2009). Dry-coated microprojection array patches for targeted delivery of immunotherapeutics to the skin. J Control Release.

[46] Edens C, Collins ML, Ayers J, Rota PA, Prausnitz MR (2013). Measles vaccination using a microneedle patch. Vaccine.

[47] Cormier M, Johnson B, Ameri M, Nyam K, Libiran L, Zhang DD (2004). Transdermal delivery of desmopressin using a coated microneedle array patch system. J Control Release.

[48] Kommareddy S, Baudner BC, Bonificio A, Gallorini S, Palladino G, Determan AS (2013). Influenza subunit vaccine coated microneedle patches elicit comparable immune responses to intramuscular injection in guinea pigs. Vaccine.

[49] Chu LY, Choi S-O, Prausnitz MR (2010). Fabrication of dissolving polymer microneedles for controlled drug encapsulation and delivery: bubble and pedestal microneedle designs. J Pharm Sci.

[50] Fukushima K, Ise A, Morita H, Hasegawa R, Ito Y, Sugioka N (2011). Two-layered dissolving microneedles for percutaneous delivery of peptide/protein drugs in rats. Pharm Res.

[51] Lee JW, Park J-H, Prausnitz MR (2008). Dissolving microneedles for transdermal drug delivery. Biomaterials.

[52] Lee K, Lee CY, Jung H (2011). Dissolving microneedles for transdermal drug administration prepared by stepwise controlled drawing of maltose. Biomaterials.

[53] Migalska K, Morrow DIJ, Garland MJ, Thakur R, Woolfson AD, Donnelly RF (2011). Laser-engineered dissolving microneedle arrays for transdermal macromolecular drug delivery. Pharm Res.

[54] Raphael AP, Prow TW, Crichton ML, Chen X, Fernando GJP, Kendall MAF (2010). Targeted, needle-free vaccinations in skin using multilayered, densely-packed dissolving microprojection arrays. Small.

[55] Sullivan SP, Koutsonanos DG, Martin MP, Lee JW, Zarnitsyn V, Choi S-O (2010). Dissolving polymer microneedle patches for influenza vaccination. Nat Med.

[56] Ito Y, Yoshimitsu J-I, Shiroyama K, Sugioka N, Takada K (2006). Self-dissolving microneedles for the percutaneous absorption of EPO in mice. J Drug Target.

[57] McGrath MG, Vucen S, Vrdoljak A, Kelly A, O’Mahony C, Moore A. Production of dissolvable microneedles using an atomised spray process: effect of microneedle composition on skin penetration. Eur J Pharm Biopharm. 2014;86:200–11. 10.1016/j.ejpb.2013.04.023 23727511

[58] Liu S, Jin M-n, Quan Y-s, Kamiyama F, Kusamori K, Katsumi H (2014). Transdermal delivery of relatively high molecular weight drugs using novel self-dissolving microneedle arrays fabricated from hyaluronic acid and their characteristics and safety after application to the skin. Eur J Pharm Biopharm.

[59] McCrudden MTC, Alkilani AZ, McCrudden CM, McAlister E, McCarthy HO, Woolfson AD (2014). Design and physicochemical characterisation of novel dissolving polymeric microneedle arrays for transdermal delivery of high dose, low molecular weight drugs. J Control Release.

[60] Luttge R, Bystrova SN, van Bennekom JG, Domanski M, Loeters PWH, Lammertink RGH, AntoniusWinnubst AJ. Integrated microneedle array and a method for manufacturing thereof, WO2009/113856 (2013).

[61] Mir J, Spoonhower J, Agostinelli JA, Demejo L, Sarbadhikari KK. Replaceable microneedle cartridge for biomedical monitoring, US 20110224515 A1 (2011).

[62] Scholten D, Stumber M, Laermer F, Feyh A. Manufacturing method for a porous microneedle array and corresponding porous microneedle array and corresponding substrate composite, United States Patent Application 20110137254 (2011).

[63] Shirkhanzadeh M. Arrays of microneedles comprising porous calcium phosphate coating and bioactive agents, WO/2003/092785 (2003).

[64] Allen M, Cros F, McAllister D, Prausnitz M, Microneedle devices and methods of manufacture and use thereof, US2010312191 (A1) (2010).

[65] Ciprian I, Luck TK, Hock TFE. Microneedles, WO2006101459 (A1) (2006).

[66] Canham LT. Transferring materials into cells porous silicon, US2004220535 (A1) (2004).

[67] Todd S, Middleton I. Microneedle device for removal of bodily fluid, GB2506010 (A) (2014).

[68] Prausnitz MR, Allen MG, Gujral I-J. Microneedle device for extraction and sensing of bodily fluids, US7344499 B1 (2008).

[69] Ji J, Tay FEH, Miao J, Iliescu C (2006). Microfabricated microneedle with porous tip for drug delivery. J Micromech Microeng.

[70] Park J-H, Choi S-O, Kamath R, Yoon Y-K, Allen MG, Prausnitz MR (2007). Polymer particle-based micromolding to fabricate novel microstructures. Biomed Microdevices.

[71] Shirkhanzadeh M (2005). Microneedles coated with porous calcium phosphate ceramics: effective vehicles for transdermal delivery of solid trehalose. J Mater Sci Mater Med.

[72] Bystrova S, Luttge R (2011). Micromolding for ceramic microneedle arrays. Microelectron Eng.

[73] Verhoeven M, Bystrova S, Winnubst L, Qureshi H, Gruijl TD, Scheper RJ (2012). Applying ceramic nanoporous microneedle arrays as a transport interface in egg plants and an ex-vivo human skin model. Microelectron Eng.

[74] Cai B, Xia W, Bredenberg S, Engqvist H (2014). Self-setting bioceramic microscopic protrusions for transdermal drug delivery. J Mater Chem B.

[75] de Groot J, Verhoeven M, Rivas DF, de Gruij TD, Scheper RJ, Luttge R. Micromolded nanoporous ceramic microneedle arrays, 2^nd^ International conference on Microneedles, 2012, Cork, Ireland.

[76] Engqvist H, Bredenberg S, Pettersson A, Lundqvist T, Pahlgren A, Sagstrom A. Transdermal drug administration device, US 2013/0273119 A1 (2013).

[77] Jäger C, Finkenberger B, Jäger W, Christophersen M, Carstensen J, Föll H (2000). Transmission electron microscopy investigations of the formation of macropores in n- and p-Si(001)/(111). Mater Sci Eng B.

[78] Salonen J, Kaukonen AM, Hirvonen J, Letho V-P (2007). Mesoporous silicon in drug delivery applications. J Pharm Sci.

[79] Low SP, Voelcker NH, Canham LT, Williams KA. The biocompatibility of porous silicon in tissues of the eye. Biomaterials. (2009). 10.1016/j.biomaterials.2009.02.008 19251317

[80] Anderson SHC, Elliott H, Wallis DJ, Canham LT, Powell JJ. Dissolution of different forms of partially porous silicon wafers under simulated physiological conditions, Phys Stat Sol. 2003;(a), 197.

[81] Klyshko A, Balucani M, Ferrari A (2008). Mechanical strength of porous silicon and its possible applications. Superlattice Microst.

[82] Paik S-J, Byun S, Lim J-M, Park Y, Lee A, Chung S (2004). In-plane single-crystal-silicon microneedles for minimally invasive microfluid systems. Sensors Actuators A Phys.

[83] McAllister DV, Wang PM, Davis SP, Park J-H, Canatella PJ, Allen MG (2003). Microfabricated needles for transdermal delivery of macromolecules and nanoparticles: fabrication methods and transport studies. Proc Natl Acad Sci U S A.

[84] Zahn JD, Talbot NH, Liepmann D, Pisano AP (2000). Microfabricated polysilicon microneedles for minimally invasive biomedical devices. Biomed Microdevices.

[85] Arcos D, Vallet-Regí M (2013). Bioceramics for drug delivery. Acta Mater.

[86] Schaefer S, Detsch R, Uhl F, Deisinger U, Ziegler G (2011). How degradation of calcium phosphate bone substitute materials is influenced by phase composition and porosity. Adv Eng Mater.

[87] Komlev VS, Mastrogiacomo M, Pereira RC, Peyrin F, Rustichelli F, Cancedda R (2010). Biodegradation of porous calcium phosphate scaffolds in an ectopic bone formation model studied by X-ray computed microtomography. Eur Cells Mater.

[88] Ding S-J, Wang C-W, Chen DC-H, Chang H-C (2005). In vitro degradation behavior of porous calcium phosphates under diametral compression loading. Ceram Int.

[89] Bose S, Darsell J, Hosick HL, Yang L, Sarkar DK, Bandyopadhyay A (2002). Processing and characterization of porous alumina scaffolds. J Mater Sci.

[90] Kim Y-H, Anirban JM, Song H-Y, Seo H-S, Lee B-T (2011). In vitro and in vivo evaluations of 3D porous TCP-coated and non-coated alumina scaffolds. J Biomater Appl.

[91] Walpole AR, Xia Z, Wilson CW, Triffitt JT, Wilshaw PR (2009). A novel nano-porous alumina biomaterial with potential for loading with bioactive materials. J Biomed Mater Res Part A.

[92] Szutkowska M. Fracture toughness of advanced alumina ceramics and alumina matrix composites used for cutting tool edges. J Achiev Mat Manuf Eng. 2012;54.

[93] Ericson F, Johansson S, Schweitz J-Å (1988). Hardness and fracture toughness of semiconducting materials studied by indentation and erosion techniques. Mater Sci Eng A.

[94] Webster TJ, Ergun C, Doremus RH, Siegel RW, Bizios R (2001). Enhanced osteoclast-like cell functions on nanophase ceramics. Biomaterials.

[95] Verbaan FJ, Bal SM, van den Berg DJ, Dijksman JA, Hecke M, Verpoorten H (2008). Improved piercing of microneedle arrays in dermatomed human skin by an impact insertion method. J Control Release.

[96] Donnelly RF, Garland MJ, Morrow DIJ, Migalska K, Singh TRR, Majithiya R (2010). Optical coherence tomography is a valuable tool in the study of the effects of microneedle geometry on skin penetration characteristics and in-skin dissolution. J Control Release.

[97] Mohammed D, Matts PJ, Hadgraft J, Lane ME (2012). Variation of stratum corneum biophysical and molecular properties with anatomic site. AAPS J.

[98] Pawlaczyk M, Lelonkiewicz M, Wieczorowski M (2013). Age-dependent biomechanical properties of the skin. Postep Derm Alergol.

[99] Sandy-Moller J, Poulsen T, Wulf HC (2003). Epidermal thickness at different body sites: relationship to age gender, pigmentation, blood content, skin type and smoking habits. Acta Derm Venereol.

[100] Waterston K, Naysmith L, Rees JL. Variation in skin thickness may explain some of the within-person variation in ultraviolet radiation-induced erythema at different body sites. J Investig Dermatol. 2005;124:1078–8. 10.1111/j.0022-202X.2005.23704.x 15854053

